# Trends in survival during the pandemic in patients with critical COVID-19 receiving mechanical ventilation with or without ECMO: analysis of the Japanese national registry data

**DOI:** 10.1186/s13054-022-04187-7

**Published:** 2022-11-15

**Authors:** Shinichiro Ohshimo, Keibun Liu, Takayuki Ogura, Yoshiaki Iwashita, Shigeki Kushimoto, Nobuaki Shime, Satoru Hashimoto, Yuji Fujino, Shinhiro Takeda, on behalf of the Japan ECMO Network

**Affiliations:** 1 Non-Profit Organization Japan ECMO Network, Tokyo, Japan; 2 Department of Emergency and Critical Care Medicine, Graduate School of Biomedical and Health Sciences, Hiroshima University, 1-2-3 Kasumi, Minami-ku, Hiroshima, 734-8551 Japan; 3 Critical Care Research Group, The Prince Charles Hospital, Chermside, Australia; 4 Tochigi Emergency and Critical Care Centre, Imperial Foundation Saiseikai, Utsunomiya Hospital, Utsunomiya, Japan; 5 Department of Emergency and Critical Care Medicine, Faculty of Medicine, Shimane University, Izumo, Japan; 6 Division of Emergency and Critical Care Medicine, Tohoku University Graduate School of Medicine, Sendai, Japan; 7 Department of Intensive Care Medicine, Kyoto Prefectural University of Medicine, Kyoto, Japan; 8 Non-Profit Organization ICU Collaboration Network (ICON), Tokyo, Japan; 9 Department of Anesthesiology and Intensive Care, Osaka University Graduate School of Medicine, Suita, Japan; 10 Kawaguchi Cardiovascular and Respiratory Hospital, Kawaguchi, Japan

**Keywords:** Acute respiratory failure, Mechanical ventilation, Coronavirus disease, Survival, Prognosis, Mortality

## Abstract

**Background:**

The survival rate of patients with critical coronavirus disease-19 (COVID-19) over time is inconsistent in different settings. In Japan, a national database was organized to monitor and share the patient generation across the country in an immediate response to the COVID-19 pandemic. This study aimed to evaluate changes in survival over time and the prognostic factors in critical COVID-19 patients receiving mechanical ventilation with/without extracorporeal membrane oxygenation (ECMO) using the largest database in Japan.

**Methods:**

This is a prospective observational cohort study of patients admitted to intensive care units in Japan with fatal COVID-19 pneumonia receiving mechanical ventilation and/or ECMO. We developed a prospective nationwide registry covering > 80% of intensive care units in Japan, and analyzed the association between patients’ backgrounds, institutional ECMO experience, and timing of treatment initiation and prognosis between February 2020 and November 2021. Prognostic factors were evaluated by Kaplan–Meier analysis and Cox proportional hazards analysis.

**Results:**

A total of 9418 patients were ventilated, of whom 1214 (13%) received ECMO. The overall survival rate for ventilated patients was 79%, 65% for those receiving ECMO. There have been five outbreaks in Japan to date. The survival rate of ventilated patients increased from 76% in the first outbreak to 84% in the fifth outbreak (*p* < 0.001). The survival rate of ECMO patients remained unchanged at 60–68% from the first to fifth outbreaks (*p* = 0.084). Age of ≥ 59 (hazard ratio [HR] 2.17; 95% confidence interval [CI] 1.76–2.68), ventilator days of ≥ 3 before starting ECMO (HR 1.91; 95% CI 1.57–2.32), and institutional ECMO experiences of ≥ 11 (HR 0.70; 95% CI 0.58–0.85) were independent prognostic factors for ECMO.

**Conclusions:**

During five COVID-19 outbreaks in Japan, the survival rate of ventilated patients tended to have gradually improved, and that of ECMO patients did not deteriorate. Older age, longer ventilator days before starting ECMO, and fewer institutional ECMO experiences may be independent prognostic factors for critical COVID-19 patients receiving ECMO.

**Supplementary Information:**

The online version contains supplementary material available at 10.1186/s13054-022-04187-7.

## Introduction

The infectious disease caused by the novel coronavirus (COVID-19), a worldwide pandemic since the end of 2019, has generated high numbers of patients with critical respiratory failure [1]. To date, various therapeutic drugs have been developed for COVID-19 pneumonia, and some appear to be effective [2–4]. However, the number of effective drugs for acute respiratory distress syndrome (ARDS) caused by critical COVID-19 is still limited [5]. Minimizing severe hypoxemia and patient self-inflicted lung injury by appropriate management with mechanical ventilation or extracorporeal membrane oxygenation (ECMO) may be effective for improving the prognosis of critical COVID-19-associated ARDS [6–8]. However, the survival rates associated with ventilatory and ECMO management in patients with critical COVID-19-associated ARDS are insufficient [9], with some studies demonstrating gradually worsening outcomes over time [10, 11]. In Japan, a national database was organized to monitor and share the patient generation across the country in an immediate response to the COVID-19 pandemic.

This study aimed to evaluate changes in survival over time and the prognostic factors in critical COVID-19 patients receiving mechanical ventilation with/without extracorporeal membrane oxygenation (ECMO) using the largest database in Japan [12].

## Materials and methods

### Development of the Japan ECMO network

In Japan, with the support of several academic societies, a non-profit organization called the Japan ECMO Network was established on February 15, 2020, to address several difficult situations [12, 13] . Specifically, we established a cooperative system of communication between high- and low-volume centers by telephone, e-mail, and direct visits. In addition, we provided training seminars on ECMO and mechanical ventilation in all 47 prefectures in Japan, and published medical information on ECMO, ventilation, and COVID-19 on our website and in articles [14, 15]. We dispatched a large number of doctors, nurses, and clinical engineers to areas where an outbreak was occurring, such as Okinawa and Tokyo, to provide medical support for a long period of time (Additional file 1: Table S1). The medical support comprised evaluation of the indications for ECMO, medical interventions associated with ECMO, ECMO transport, and instructions on appropriate ventilatory management and prone positioning for moderately ill patients.

### Study settings

We developed a novel web-based database system, CRoss Icu Searchable Information System (CRISIS), to properly track real-time information in ICUs throughout Japan during the COVID-19 pandemic. The research plan to collect data on several clinical variables included in CRISIS from nationwide ICUs and to use them for analysis as Japanese epidemiological data was approved by the Ethical Review Committee of Hiroshima University (approval number: E-1965). Because only publicly available data were used in this study, the need for institutional review board approval and patient consent at each institution was waived. The ethics committees of the Japanese Society of Intensive Care Medicine, the Japanese Association for Acute Medicine, and the Japanese Society of Respiratory Care Medicine agreed to this waiver. The total number of ICU beds registered in CRISIS is > 6600, which covers almost all designated ICU beds including tertiary emergency centers in Japan. Since there is no officially approved definition of an ECMO center in Japan, we included in our analysis ICUs that are registered as certified facilities with these societies. These certified facilities are staffed by physicians who have acquired sufficient skills and passed the certification examinations. The included patients were patients of all ages and both sexes with critical COVID-19 who received mechanical ventilation and ECMO in Japan. Since data entry into CRISIS was not a mandatory requirement for the facilities certified by the academic societies, a 100% coverage rate could not be achieved at the start of this project. However, the number of facilities registered in CRISIS has now increased to more than 6600, which covers almost all the facilities certified by the academic societies. Including the newly added centers, there was little information regarding patients treated with mechanical ventilators and ECMO during the study period. These findings suggested that the CRISIS used in the analysis included almost all patients with critical COVID-19 who received intensive care during the five outbreaks. COVID-19 patients were defined as those with a positive severe acute respiratory syndrome coronavirus-2 (SARS-CoV-2)—polymerase chain reaction (PCR) test and pulmonary involvements typical of COVID-19.

### ECMO indications

The indications for ECMO in patients with COVID-19 generally followed the respiratory severity criteria of the EOLIA trial [16], although a standardized protocol was not used throughout Japan. However, considering the balance between medical resources and patient survival, the Japan ECMO network released nationwide information that ECMO could be considered in cases of progressive deterioration with a ratio of the partial pressure of arterial oxygen (PaO_2_)/fraction of inspired oxygen (F_i_O_2_) < 100, even with a positive end-expiratory pressure (PEEP) of ≥ 10 cm H_2_O.

In Japan, the ECMO Project Committee, established by the Japanese Society of Respiratory Medicine and the Japanese Society of Intensive Care Medicine, has regularly held educational workshops on the indications and adequate use of ECMO throughout the country since 2009. Since most institutions started using ECMO after staff attended this workshop, it is presumed that there is no significant difference in the criteria and contraindications for the use of ECMO throughout Japan.

### Data collection

Data registered in CRISIS comprise patient background characteristics, severity of acute respiratory failure, number of days of mechanical ventilation and ECMO, mortality, other adjunctive therapies, and other data. Detailed information was collected separately as text information. The data used in the analyses in this study were obtained from February 2020 to November 2021. Construction of the registry and checking for incomplete data entry were performed by non-profit organization ICU Collaboration Network (ICON). Data extraction and cleaning were performed by a different person than the person in charge of the data analysis.

### Statistical analysis

Patient characteristics data were expressed as *n* (%) for categorical variables and as median (interquartile range, IQR) for continuous parameters, as appropriate. The chi-square test or Fisher's exact probability test was used for analysis of categorical variables, and the Mann–Whitney *U* test or multiple comparison test with Bonferroni correction was used for comparisons of continuous parameters. *p* < 0.05 was considered to indicate a significant difference. Receiver operating characteristic (ROC) curves were used to determine the cutoff values for continuous parameters. The Kaplan–Meier method and Cox proportional hazards model were used for survival time analysis. We selected items with a *p*-value < 0.05 in univariate analysis as variables to be included in the model. Univariate and multivariate analyses were conducted with continuous variables either as they were, or as nominal variables bisected by a cutoff value. In model 1, as many continuous variables as possible were included in the multivariate analysis. In model 2, as many nominal variables as possible were included in the multivariate analysis, bisected by cutoff values. All statistical analyses were performed using R software (R Foundation for Statistical Computing, Vienna, Austria) and SPSS (IBM Corporation, Armonk, NY).

## Results

### Patient characteristics

Table 1 shows the patients’ characteristics. A total of 9418 patients were enrolled in the registry; 8204 (87%) received mechanical ventilation alone and 1214 (13%) received ECMO. Compared with the ventilator-alone group, the ECMO group was predominantly male, younger, and had a higher BMI (all *p*-values < 0.001). The PaO_2_/F_i_O_2_ ratio before starting ECMO was 85 (IQR, 70–104).

**Table 1:** Patient characteristics

Variable	Total cohort	Ventilator alone group	ECMO group	*p*-value*
Number of patients	9418	8204	1214	
Male gender	7093 (75)	6176 (75)	971 (80)	** < 0.001**
Age	64 (54–73)	65 (55–74)	58 (50–65)	** < 0.001**
Height (cm)	167 (160–172)	166 (160–171)	169 (163–173)	** < 0.001**
Weight (kg)	70 (61–82)	70 (60–80)	79 (69–90)	** < 0.001**
Body mass index (kg/m^2^)	25.7 (22.9–29.1)	25.4 (22.8–28.5)	27.8 (24.7–31.6)	** < 0.001**
PaO_2_/F_I_O_2_ at starting ECMO	N/A	N/A	85 (70–104)	N/A
PEEP at starting ECMO (cmH2O)	N/A	N/A	12 (10–15)	N/A

Bold indicates analyses with statistically significant differences (*p* < 0.05)

Data are expressed as number (percentage) or median (interquartile range, IQR)

N/A, not available

*The *p*-values are the values comparing the ventilation alone group with the ECMO group

### Trends in the number of patients and the proportion of patients with ECMO added to mechanical ventilation

Additional file 2: Fig. S1 shows the number of new COVID-19 patients per week. To date, there have been five COVID-19 outbreaks in Japan. In the first outbreak, the percentage of patients who used ECMO was high at over 30%; however, this percentage decreased gradually thereafter and remained unchanged at approximately 10–15% in the second to fifth outbreaks.

### Trends in the overall survival rate

The overall survival rate was 77%; 79% in the ventilator-alone group and 65% in the ECMO group (*p* < 0.001, Table 2). The 90-day survival rates were similar. The number of patients receiving mechanical ventilation increased with each outbreak from the first to the fifth outbreaks (Additional file 3: Fig. S2A). However, the survival rate, which was approximately 70% during the first outbreak, improved continuously to approximately 80% in the fifth outbreak. In the third outbreak, the survival rate of patients receiving ECMO decreased temporarily to 57%, but in the remainder of the outbreaks, survival remained almost unchanged at 63–68% (Additional file 3: Fig: S2B). The survival rate multiplied by the number of patients per month (busyness-adjusted survival index) improved continuously from 26.0 in the first outbreak to 57.6 in the fifth outbreak.

**Table 2:** Patient outcome

Variable	Total cohort	Ventilator alone group	ECMO group	*p*-value*
Ventilator days before starting ECMO (days)	N/A	N/A	1 (0–5)	N/A
Ventilator days (days)	10 (6–19)	9 (6–16)	24 (15–44)	** < 0.001**
ECMO days (days)	N/A	N/A	13 (9–24)	N/A
Overall survival	7239 (77)	6515 (79)	785 (65)	** < 0.001**
90-day survival	7265 (77)	6524 (80)	801 (66)	** < 0.001**

Bold indicates analyses with statistically significant differences (*p* < 0.05)

Data are expressed as number (percentage) or median (interquartile range, IQR)

N/A, not available

*The *p*-values are the values comparing the ventilation alone group with the ECMO group

### Association between ventilator days before starting ECMO and survival

**Fig. 1:**
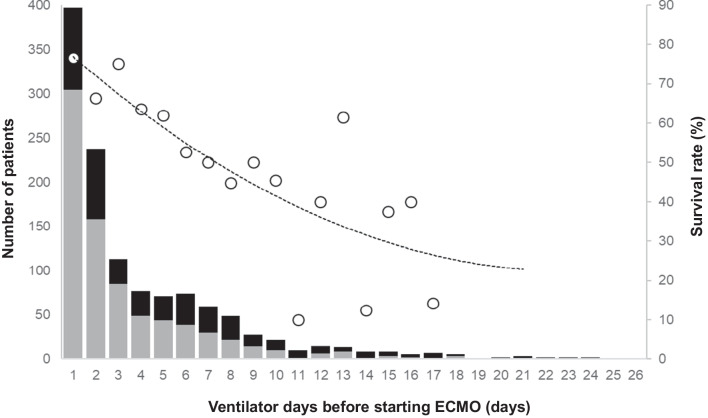
Ventilator days before starting ECMO and survival rate. The majority of patients were changed to ECMO after 2–3 ventilator days; however, some patients were changed to ECMO after a longer period of ventilatory management. The survival rate decreased gradually in accordance with a higher number of ventilator days before starting ECMO. The gray bars indicate the numbers of survivors, the black bars indicate the numbers of deaths, and the white circles indicate the survival rates. ECMO, extracorporeal membrane oxygenation

Figure 1 shows the association between the number of ventilator days before starting ECMO and the survival rate. The survival rate decreased as the number of ventilator days before starting ECMO increased. When the number of ventilator days was > 6, the survival rate decreased to < 50%. The majority of patients were started ECMO within 6 days, while the remaining patients were started ECMO after longer ventilatory management.

### Association between the number of outbreaks and the survival rate

**Fig. 2:**
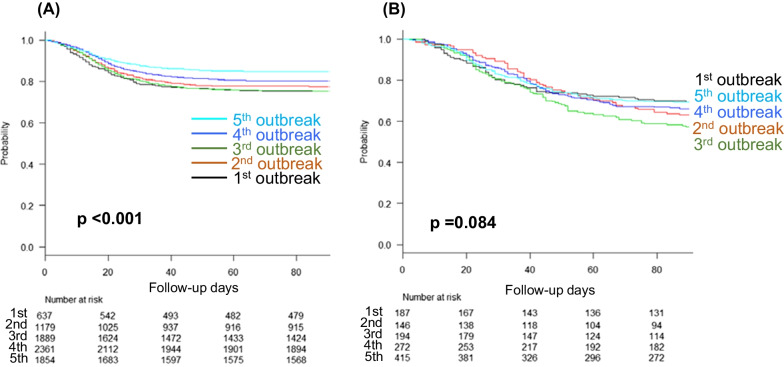
Survival curves for the five COVID-19 outbreaks to date. **A** Survival curve of patients receiving mechanical ventilation, showing gradual improvement in survival from the first outbreak to the fifth outbreak. In the *post hoc* analysis, there was a significant difference between the first and fourth outbreaks (*p* = 0.033) and between the third and fourth outbreaks (*p* = 0.001). There was also a significant difference between the fifth outbreak and all other outbreaks (all *p* < 0.001). **B** Survival curve of patients receiving ECMO, showing that the survival rate remained nearly the same from the first outbreak to the fifth outbreak. COVID-19, coronavirus disease 2019; ECMO, extracorporeal membrane oxygenation

Figure 2 shows the association between the number of outbreaks and the survival curves of critical COVID-19 patients receiving mechanical ventilation and ECMO. The survival rate of ventilated patients was the lowest during the first and third outbreaks, which improved in the fourth and the fifth outbreaks (*p* < 0.001). In contrast, the survival rate of patients receiving ECMO was almost constant from the first to the fifth outbreaks, and there was no significant difference between all groups in the first through fifth outbreaks (*p* = 0.084).

### Survival curve analysis of patients receiving mechanical ventilation

**Fig. 3:**
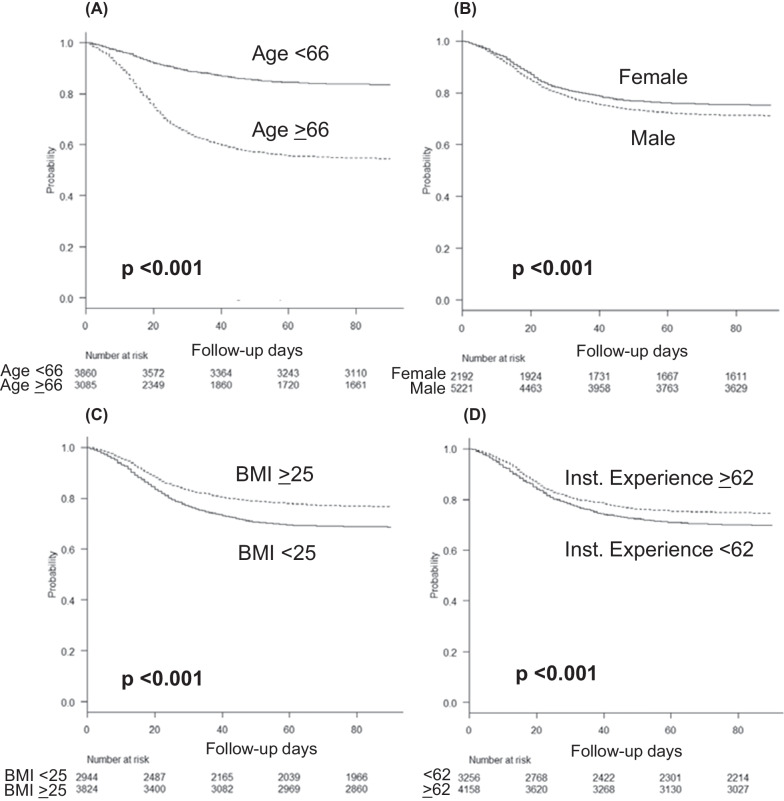
Survival curve analysis for predicting mortality in patients with severe COVID-19 receiving mechanical ventilation. **A** Survival curve for age, with a cutoff of 66 years; **B** for sex; **C** for body mass index, with a cutoff of 25; and **D** for the number of mechanical ventilations experienced at an institution for patients with severe COVID-19, with a cutoff of 62 cases. COVID-19, coronavirus disease 2019

Additional file 4: Fig. S3 shows the results of the ROC curve analysis of the predictors of mortality in critical COVID-19 patients receiving mechanical ventilation. The areas under the ROC curve (AUC) and 95% confidence intervals (CIs) for each parameter were as follows: age, 0.71 (0.70–0.72); BMI, 0.57 (0.55–0.58); ventilatory days, 0.74 (0.73–0.76); and the number of institutional treatment experience of COVID-19 patients requiring mechanical ventilations, 0.52 (0.51–0.54). On the basis of the ROC curve analysis, the point with the highest sensitivity and specificity was set as the optimal cutoff value. Age > 66 years, male sex, BMI < 25, and the number of institutional treatment experience of COVID-19 patients requiring mechanical ventilations less than 62 were associated with a significantly decreased survival rate (all *p*-values < 0.001, Fig. 3).

### Survival curve analysis of patients receiving ECMO

**Fig. 4:**
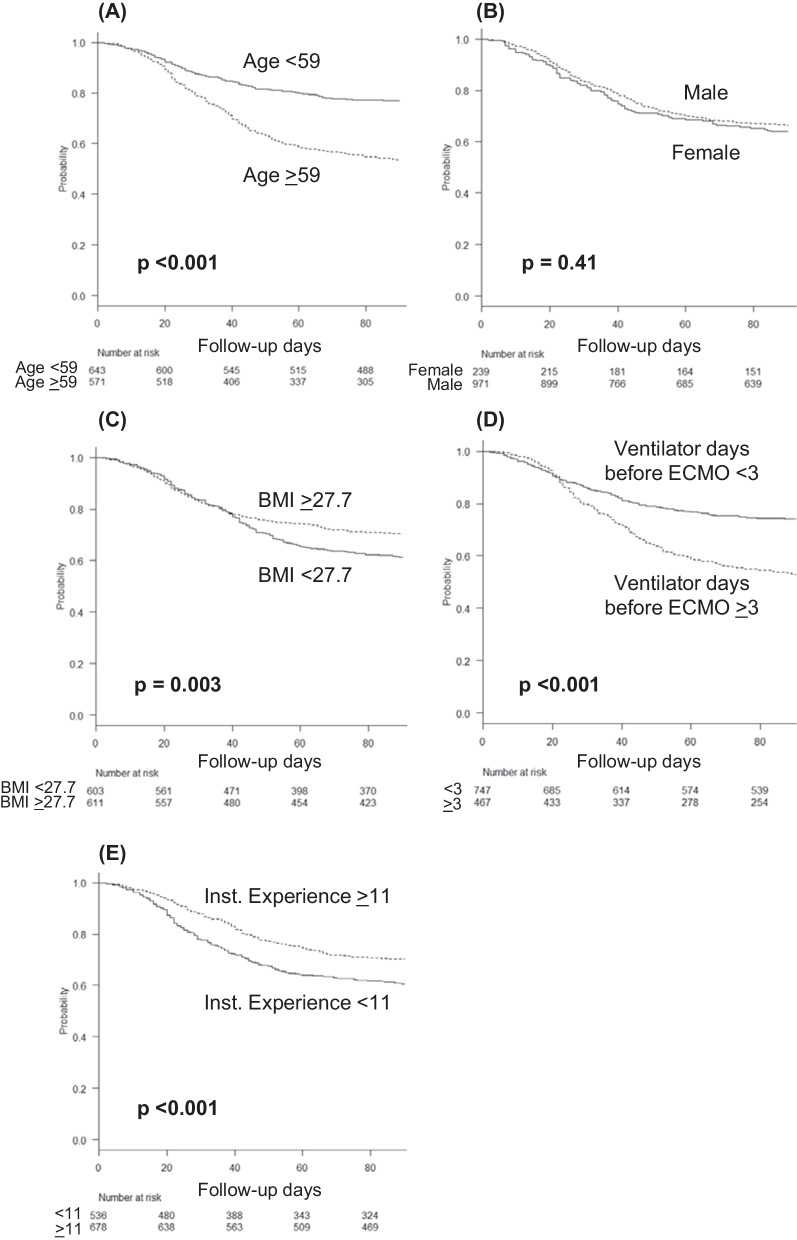
Survival curve analysis for predicting mortality in patients with critical COVID-19 receiving ECMO. **A** Survival curve for age, with a cutoff of 59 years; **B** for sex; **C** for body mass index, with a cutoff of 27.7; **D** for the number of ventilator days before starting ECMO, with a cutoff of 3; and **E** for the number of ECMO experiences at an institution for patients with critical COVID-19, with a cutoff of 11. COVID-19, coronavirus disease 2019; ECMO, extracorporeal membrane oxygenation

Additional file 5: Fig. S4 shows the results of the ROC curve analysis for predicting mortality in patients with critical COVID-19 receiving ECMO. The AUCs and 95% CIs for each variable were as follows: age, 0.67 (0.64–0.70); BMI, 0.57 (0.53–0.60); ventilatory days before starting ECMO, 0.64 (0.61–0.67); number of ECMO cases experienced at an institution, 0.56 (0.52–0.59); and number of ECMO days, 0.68 (0.65–0.71). Age ≥ 59 years (*p* < 0.001), BMI < 27.7 (*p* = 0.003), ≥ 3 ventilatory days before starting ECMO (*p* < 0.001), and < 11 ECMO experiences at an institution (*p* < 0.001) were significantly associated with mortality; sex was not a prognostic factor (Fig. 4).

### Poor prognostic factors in ventilated patients and ECMO patients

Tables 3 and 4 show the univariate and multivariate analyses of poor prognostic factors in critical COVID-19 patients receiving mechanical ventilation and ECMO. Among the several candidate predictors identified in the univariate analysis, age (continuous variable or above the cutoff of 66 years), BMI (continuous variable), and institutional treatment experience of COVID-19 patients requiring mechanical ventilations < 62 were the independent predictors. In contrast, in patients with critical COVID-19 receiving ECMO, age (continuous variable or above the cutoff of 59 years), ≥ 3 ventilator days before starting ECMO, and institutional experience of ECMO (continuous variable or above the cutoff of 11 cases) were the independent poor prognostic factors.

**Table 3:** Univariate and multivariate analyses for predicting poor outcome in patients with COVID-19 receiving mechanical ventilation

Univariate analysis				
Variable	HR	95% CI (Lower)	95% CI (Upper)	*p*-value
Age (cont.)	1.05	1.05	1.06	** < 0.001**
Age ≥ 66	2.10	2.65	3.19	** < 0.001**
Male gender	0.88	0.79	0.97	**0.008**
BMI (cont.)	0.97	0.96	0.98	** < 0.001**
BMI ≥ 25.0	0.74	0.67	0.81	** < 0.001**
Inst. experience of mechanical ventilation (cont.)	1.00	1.00	1.00	0.19
Inst. experience of mechanical ventilation ≥ 62	0.81	0.74	0.88	** < 0.001**
COVID-19 outbreak (fifth vs others)	0.90	0.87	0.93	** < 0.001**
*Multivariate analysis (Model 1)*				
Age (cont.)	1.06	1.06	1.07	** < 0.001**
Male gender	1.08	0.97	1.20	0.14
BMI (cont.)	1.03	1.02	1.04	** < 0.001**
Inst. experience of mechanical ventilation ≥ 62	0.81	0.74	0.89	** < 0.001**
COVID-19 outbreak (fifth vs others)	1.20	0.98	1.46	0.76
*Multivariate analysis (Model 2)*				
Age ≥ 66	3.03	2.70	3.39	** < 0.001**
Male gender	0.99	0.89	1.10	0.85
BMI ≥ 25.0	0.99	0.90	1.09	0.84
Inst. experience of mechanical ventilation ≥ 62	0.81	0.74	0.88	** < 0.001**
COVID-19 outbreak (fifth vs others)	1.10	0.90	1.35	0.34

Bold indicates analyses with statistically significant differences (*p* < 0.05)

*HR* hazard ratio; *CI* confidence interval; *BMI* body mass index; *ECMO* extracorporeal membrane oxygenation; *COVID-19* coronavirus disease 2019; *P/F* partial pressure of arterial oxygen/fraction of inspiratory oxygen; *PEEP* positive end-expiratory pressure; *Cont.* continuous; *Inst.* institutional

**Table 4:** Univariate and multivariate analyses for predicting poor outcome in patients with COVID-19 receiving ECMO

Univariate analysis				
Variable	HR	95% CI (Lower)	95% CI (Upper)	*p*-value
Age (cont.)	1.05	1.04	1.06	** < 0.001**
Age ≥ 59	2.31	1.89	2.83	** < 0.001**
Male gender	0.91	0.71	1.15	0.41
BMI (cont.)	0.99	0.97	1.01	0.20
BMI ≥ 27.7	0.75	0.61	0.91	**0.003**
Ventilator days before ECMO (cont.)	1.04	0.87	1.24	0.67
Ventilator days before ECMO ≥ 3	2.01	1.65	2.44	** < 0.001**
Inst. experience of ECMO (cont.)	0.99	0.98	1.00	**0.003**
Inst. experience of ECMO ≥ 11	0.69	0.57	0.83	** < 0.001**
P/F ratio at starting ECMO	1.00	1.00	1.00	0.77
PEEP at starting ECMO	1.00	1.00	1.00	0.59
COVID-19 outbreak (fifth vs others)	1.00	0.73	1.36	0.98
*Multivariate analysis (Model 1)*				
Age (cont.)	1.04	1.03	1.05	** < 0.001**
BMI ≥ 27.7	1.06	0.87	1.30	0.56
Ventilator days before ECMO ≥ 3	1.82	1.50	2.21	** < 0.001**
Inst. experience of ECMO (cont.)	0.99	0.98	1.00	**0.015**
*Multivariate analysis (Model 2)*				
Age ≥ 59	2.17	1.76	2.68	** < 0.001**
BMI ≥ 27.7	1.02	0.84	1.26	0.82
Ventilator days before ECMO ≥ 3	1.91	1.57	2.32	** < 0.001**
Inst. experience of ECMO ≥ 11	0.70	0.58	0.85	** < 0.001**

Bold indicates analyses with statistically significant differences (*p* < 0.05)

*HR* hazard ratio; *CI* confidence interval; *BMI* body mass index; *ECMO* extracorporeal membrane oxygenation; *COVID-19* coronavirus disease 2019; *P/F* partial pressure of arterial oxygen/fraction of inspiratory oxygen; *PEEP* positive end-expiratory pressure; *cont*. continuous; *Inst*. institutional

## Discussion

This study showed the incidence and outcomes of critical COVID-19-associated ARDS from an epidemiological perspective using a nationwide registry covering most of the ICUs in Japan. Although the number of ventilated patients per month has spiked five times to date, the rate of ECMO introduction has decreased over time. The survival rate of patients receiving mechanical ventilation tended to improve over the study period, and that of patients receiving ECMO remained almost constant, with no significant deterioration over time in either group. The independent poor prognostic factors for ECMO patients were older age, longer ventilatory days before starting ECMO, and lower number of institutional experiences of ECMO.

Previous studies [17, 18] have showed a survival rate of 50–52% for COVID-19 patients who received ECMO. In a meta-analysis involving 22 studies with data from Japan, the survival rate with ECMO in COVID-19 was 64% [19]. However, many of the studies included in this meta-analysis were small, and the survival rate varied greatly, ranging from 35 to 85%. The PaO_2_/F_I_O_2_ ratio and PEEP before starting ECMO in Japan were almost the same as recommended by EuroELSO. Even though the median age was 61 years, slightly older than that in the EuroELSO cohort, the survival rate of 68% in Japan appeared to be comparable to that in other countries.

Several previous studies [10, 11, 20] found that survival rates in patients with critical COVID-19 who received ECMO may have gradually declined over time. This decreased survival rate may have been associated with prolonged high-flow therapy and failure of noninvasive positive pressure ventilation prior to tracheal intubation [21, 22]. This was because noninvasive respiratory support may increase the severity of patient self-inflicted lung injury if excessive spontaneous breathing was not adequately controlled [23, 24]. There may have been multiple factors involved in these longitudinal declines in outcomes, including ECMO indication criteria, centralization, changes in treatment protocols, and well-developed vaccine. Although the strength of these confounding factors in Japan was unknown, the unchanged or slightly improved outcomes in Japan appeared to be a novelty.

Older age was a poor prognostic factor in patients with critical COVID-19-associated ARDS who received ECMO in Japan, consistent with previous studies [25]. The significant decrease in survival rate with each additional ventilatory day prior to ECMO initiation suggested the importance of timely decision-making for ECMO indication. Although ECMO use should be carefully considered because of the risks of various complications, an appropriate balance between the advantages and disadvantages regarding the time to decision would be necessary.

Previous studies [26, 27] showed that annual experience of > 30 cases appeared to be associated with an improved survival rate. Although our analysis indicated a low cutoff of 11 total cases, the number of ECMO experiences at an institution was significantly associated with improved patient survival. However, during the COVID-19 pandemic, the number of patients dramatically increased, making intensive care at ECMO centers difficult. In addition, Japan had problems with patient transport across different administrative regions (prefectures) and difficulties in transporting patients by air. In fact, only 156 (13%) received ECMO transport, of which only 65 (5%) received primary or secondary ECMO transport. However, this ECMO network system, information provision, and educational system may have contributed to appropriate ECMO application decisions and relatively good survival rate in critical COVID-19 patients in Japan, even in situations of incomplete centralization.

We recognize our study has the following potential limitations: First, because this study was an analysis of data from a national registry, detailed clinical information on individual patients was unavailable. Lack of information on various potential confounders was a major limitation of the study. Second, the decision to initiate or terminate mechanical ventilation or ECMO was left to the judgment of the attending physician, and no standardized protocols were used. We speculate that the inter-facility differences in ECMO indications were small, as many centers refer to protocols published by academic societies [28]. However, this lack of standardized protocols could have been a potential bias. Third, we could not directly confirm an association between the contribution of the Japan ECMO Network and changes in patient outcomes or ECMO indications. The Japan ECMO Network has developed a cooperation between high- and low-volume facilities, held training seminars, and published medical information [14, 15]. All these efforts appeared to have been comprehensively effective, and indirectly contributed to the improvement of ventilatory management techniques and the appropriateness of ECMO indications during five outbreaks. However, a prospective study would be needed to verify this causal relationship. Fourth, we could not compare outcomes before and after the establishment of the Japan ECMO Network in the COVID-19 pandemic, as CRISIS is a database created by the Japan ECMO Network after its establishment. A pre- and post-comparison of survival rates in facilities supported by the Japan ECMO Network and those not supported by the Japan ECMO Network may provide an indirect but useful assessment of the effectiveness of the Japan ECMO Network. Future research is needed to verify the direct effects of the Japan ECMO Network.

In conclusion, we showed that the survival rate of critical COVID-19-associated ARDS in Japan may be comparable to that previously reported. In addition, outcomes with mechanical ventilation tended to improve with time, and outcomes with ECMO did not deteriorate. However, older age, higher number of ventilatory days before starting ECMO, and the number of institutional ECMO experiences may be independent poor prognostic factors for patients receiving ECMO. CRISIS is one of the largest databases in Japan, covering almost all ICUs at major facilities in Japan [12]. Analysis of CRISIS appears to be the most sensitive and universal indicator of the outcome of critical COVID-19 patients in Japan. The novelty of CRISIS was not only its high data coverage, but also the real-time updating of data and the continuous feedback of the summarized data to ICU physicians in Japan, even during the pandemic. However, the available data items in CRISIS are not sufficiently comprehensive, and many unmeasured confounding factors may be relevant. Therefore, further analyses are desirable, considering sufficient confounding factors. Ideally, ECMO should probably be started promptly at a high-volume ECMO center in patients who meet the criteria for ECMO**.**

## Supplementary Information

**Additional file 1: Table 1.** Activities of the Japan ECMO network

**Additional file 2: Fig. S1.** Serial changes in the number of patients with severe COVID-19 receiving mechanical ventilation and ECMO, and the serial proportion of patients who had ECMO added to mechanical ventilation. There have been five outbreaks of COVID-19 in Japan to date, and the number of patients receiving mechanical ventilation increased continuously. However, the proportion of patients changed from mechanical ventilation to ECMO decreased continuously. The gray bars indicate the numbers of patients receiving mechanical ventilation, the black bars indicate the numbers of patients receiving ECMO, and the white circles indicate the proportions of patients who were changed from mechanical ventilation to ECMO. COVID-19, coronavirus disease 2019; ECMO, extracorporeal membrane oxygenation

**Additional file 3: Fig. S2.** Serial changes in the survival rates of patients with severe COVID-19 receiving mechanical ventilation (A) and ECMO (B). (A) The number of patients receiving mechanical ventilation increased with each outbreak from the first to the fifth outbreaks; however, the survival rate improved continuously. The gray bars indicate the numbers of survivors, the black bars indicate the numbers of deaths, and the white circles indicate the survival rates. (B) The survival rate remained nearly unchanged throughout the five outbreaks, except for a slight decrease during the third outbreak. The survival rate divided by the average number of patients per month (busyness-adjusted survival index) improved continuously. The gray bars indicate the numbers of survivors, the black bars indicate the numbers of deaths, the white circles indicate the survival rates, and the white diamonds indicate the busyness-adjusted survival index values. COVID-19, coronavirus disease 2019; ECMO, extracorporeal membrane oxygenation

**Additional file 4: Fig. S3.** ROC curve analysis for predicting poor outcome in patients with severe COVID-19 receiving mechanical ventilation. (A) ROC curve for age, (B) for body mass index, (C) for the number of ventilator days, and (D) for the number of mechanical ventilations experienced at an institution for patients with severe COVID-19. ROC, receiver operating characteristic curve; COVID-19, coronavirus disease 2019

**Additional file 5: Fig. S4.** ROC curve analysis for predicting poor outcome in patients with critical COVID-19 receiving ECMO. (A) ROC curve for age, (B) body mass index, (C) number of ventilator days before starting ECMO, (D) number of ECMO experiences at an institution for patients with critical COVID-19, and (E) number of ECMO days. ROC, receiver operating characteristic curve; COVID-19, coronavirus disease 2019; ECMO, extracorporeal membrane oxygenation

## Data Availability

Non-profit organization Japan ECMO Network. URL: https://www.ecmonet.jp/crisis.

## Abbreviations

ARDS: Acute respiratory distress syndrome
AUC: Area under the curve
BMI: Body mass index
CI: Confidence interval
COVID-19: Coronavirus disease 2019
CRISIS: CRoss Icu Searchable Information System
ECMO: Extracorporeal membrane oxygenation
ELSO: Extracorporeal Life Support Organization
F_I_O_2_: Fraction of inspired oxygen
ICU: Intensive care unit
IQR: Interquartile range
PaO_2_: Partial pressure of arterial oxygen
PCR: Polymerase chain reaction
PEEP: Positive end-expiratory pressure
ROC: Receiver operating characteristic
SARS-CoV-2: Severe acute respiratory syndrome coronavirus-2
