# Supplementary material for: Trends in survival during the pandemic in patients with critical COVID-19 receiving mechanical ventilation with or without ECMO: analysis of the Japanese national registry data
Source: Crit Care. 2022 Nov 15;26:354. doi: 10.1186/s13054-022-04187-7 (PMC9664428; doi:10.1186/s13054-022-04187-7)
Supplement: Supplementary file 1 — Additional file 1: Table 1. Activities of the Japan ECMO network [file 13054_2022_4187_MOESM1_ESM.docx]

**Supplemental Table 1. Activities of the Japan ECMO network**

| Dispatching medical staff to pandemic areas (Okinawa) | |  |
| --- | --- | --- |
|  | Period | Jun 5 to 30, 2021 |
|  | Total number of doctors | 118 |
|  | Total number of nurses | 61 |
|  | Total numberof clinical engineers | 25 |
|  | Total person-days worked | 204 |
| Dispatching medical staff to pandemic areas (Tokyo) | |  |
|  | Period | Aug 23 to Sep 17, 2021 |
|  | Total number of doctors | 267 |
|  | Total number of nurses | 22 |
|  | Total numberof clinical engineers | 12 |
|  | Total person-days worked | 301 |
| ECMO transport, n (%) | | 156 |
|  | Conventional | 91 (58) |
|  | Primary | 40 (26) |
|  | Secondary | 25 (16) |
| Number of telephone consultations (times) | | 341 |
| Communication between members via e-mail (times) | | 4,564 |
