# Supplementary material for: Trends in survival during the pandemic in patients with critical COVID-19 receiving mechanical ventilation with or without ECMO: analysis of the Japanese national registry data
Source: Crit Care. 2022 Nov 15;26:354. doi: 10.1186/s13054-022-04187-7 (PMC9664428; doi:10.1186/s13054-022-04187-7)
Supplement: Supplementary file 2 — Additional file 2: Fig. S1. Serial changes in the number of patients with severe COVID-19 receiving mechanical ventilation and ECMO, and the serial proportion of patients who had ECMO added to mechanical ventilation. There have been five outbreaks of COVID-19 in Japan to date, and the number of patients receiving mechanical ventilation increased continuously. However, the proportion of patients changed from mechanical ventilation to ECMO decreased continuously. The gray bars indicate the numbers of patients receiving mechanical ventilation, the black bars indicate the numbers of patients receiving ECMO, and the white circles indicate the proportions of patients who were changed from mechanical ventilation to ECMO. COVID-19, coronavirus disease 2019; ECMO, extracorporeal membrane oxygenation [file 13054_2022_4187_MOESM2_ESM.pptx]

## Slide 1
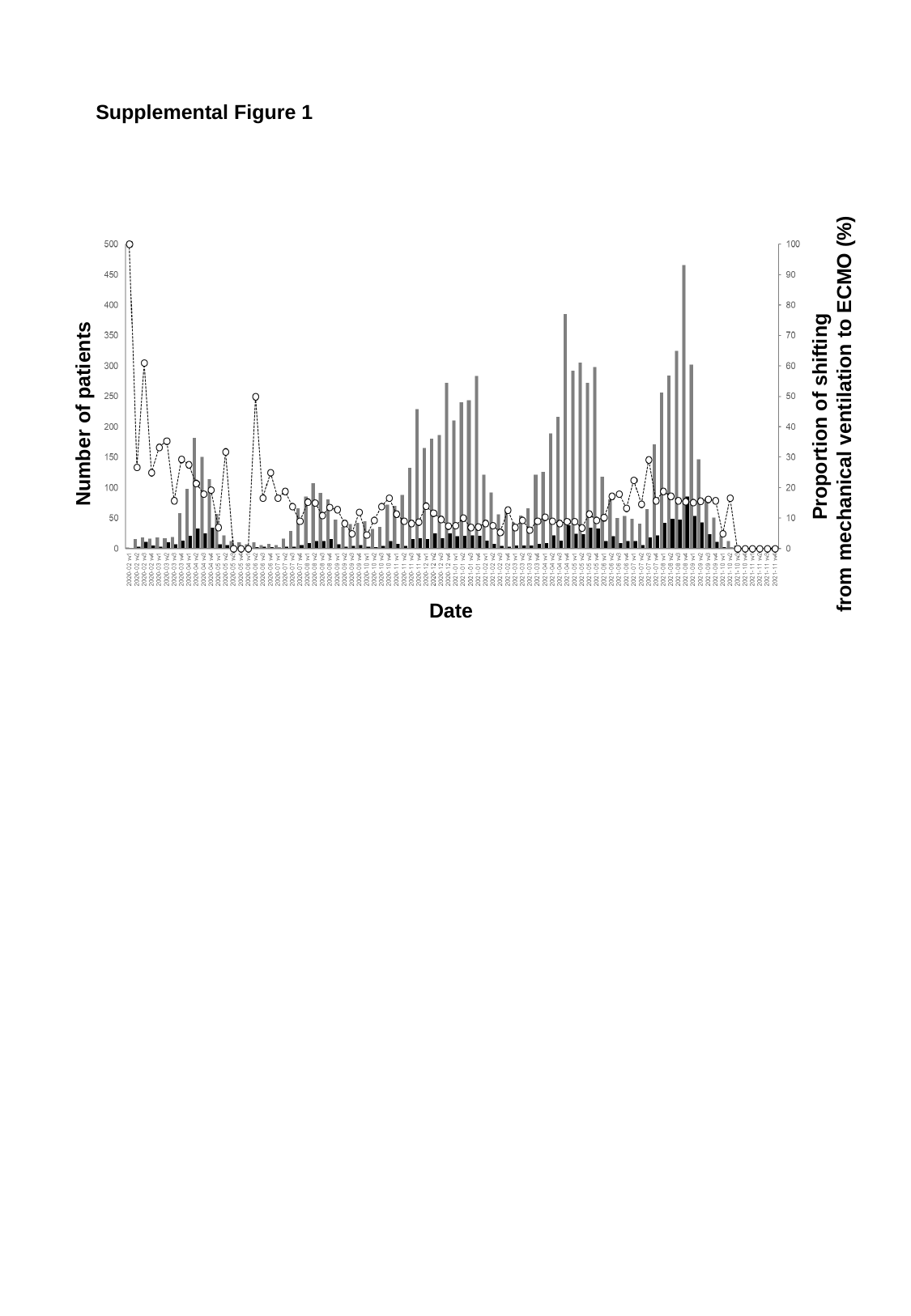

Supplemental Figure 1
Proportion of shifting
from mechanical ventilation to ECMO (%)
Number of patients
Date
