# Supplementary material for: Trends in survival during the pandemic in patients with critical COVID-19 receiving mechanical ventilation with or without ECMO: analysis of the Japanese national registry data
Source: Crit Care. 2022 Nov 15;26:354. doi: 10.1186/s13054-022-04187-7 (PMC9664428; doi:10.1186/s13054-022-04187-7)
Supplement: Supplementary file 3 — Additional file 3: Fig. S2. Serial changes in the survival rates of patients with severe COVID-19 receiving mechanical ventilation (A) and ECMO (B). (A) The number of patients receiving mechanical ventilation increased with each outbreak from the first to the fifth outbreaks; however, the survival rate improved continuously. The gray bars indicate the numbers of survivors, the black bars indicate the numbers of deaths, and the white circles indicate the survival rates. (B) The survival rate remained nearly unchanged throughout the five outbreaks, except for a slight decrease during the third outbreak. The survival rate divided by the average number of patients per month (busyness-adjusted survival index) improved continuously. The gray bars indicate the numbers of survivors, the black bars indicate the numbers of deaths, the white circles indicate the survival rates, and the white diamonds indicate the busyness-adjusted survival index values. COVID-19, coronavirus disease 2019; ECMO, extracorporeal membrane oxygenation [file 13054_2022_4187_MOESM3_ESM.pptx]

## Slide 1
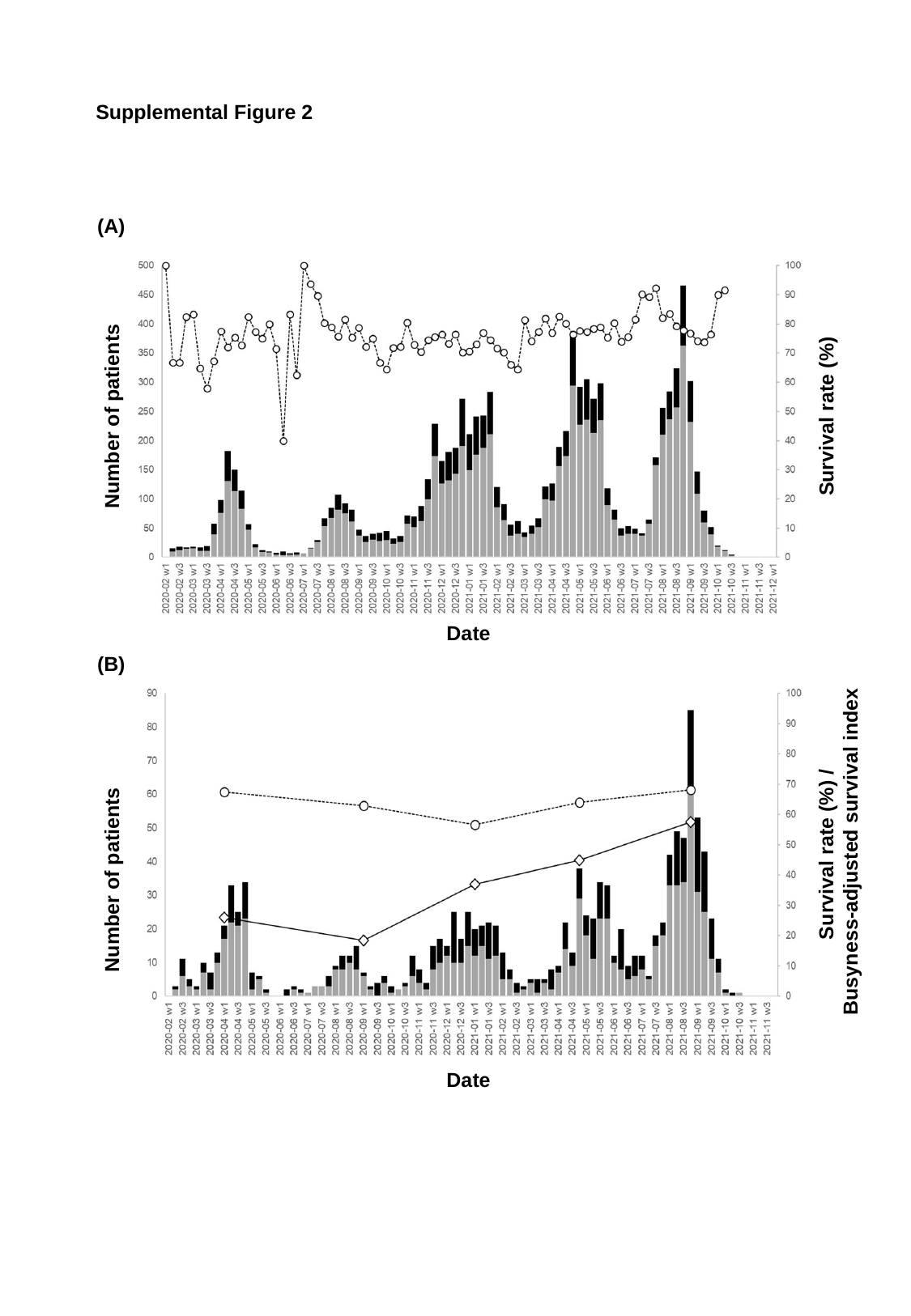

Supplemental Figure 2
(A)
Number of patients
Survival rate (%)
Date
(B)
Survival rate (%) /
Busyness-adjusted survival index
Number of patients
Date
