# Supplementary figures and images for: Trends in survival during the pandemic in patients with critical COVID-19 receiving mechanical ventilation with or without ECMO: analysis of the Japanese national registry data
Source: Crit Care. 2022 Nov 15;26:354. doi: 10.1186/s13054-022-04187-7 (PMC9664428; doi:10.1186/s13054-022-04187-7)

## Slide 1
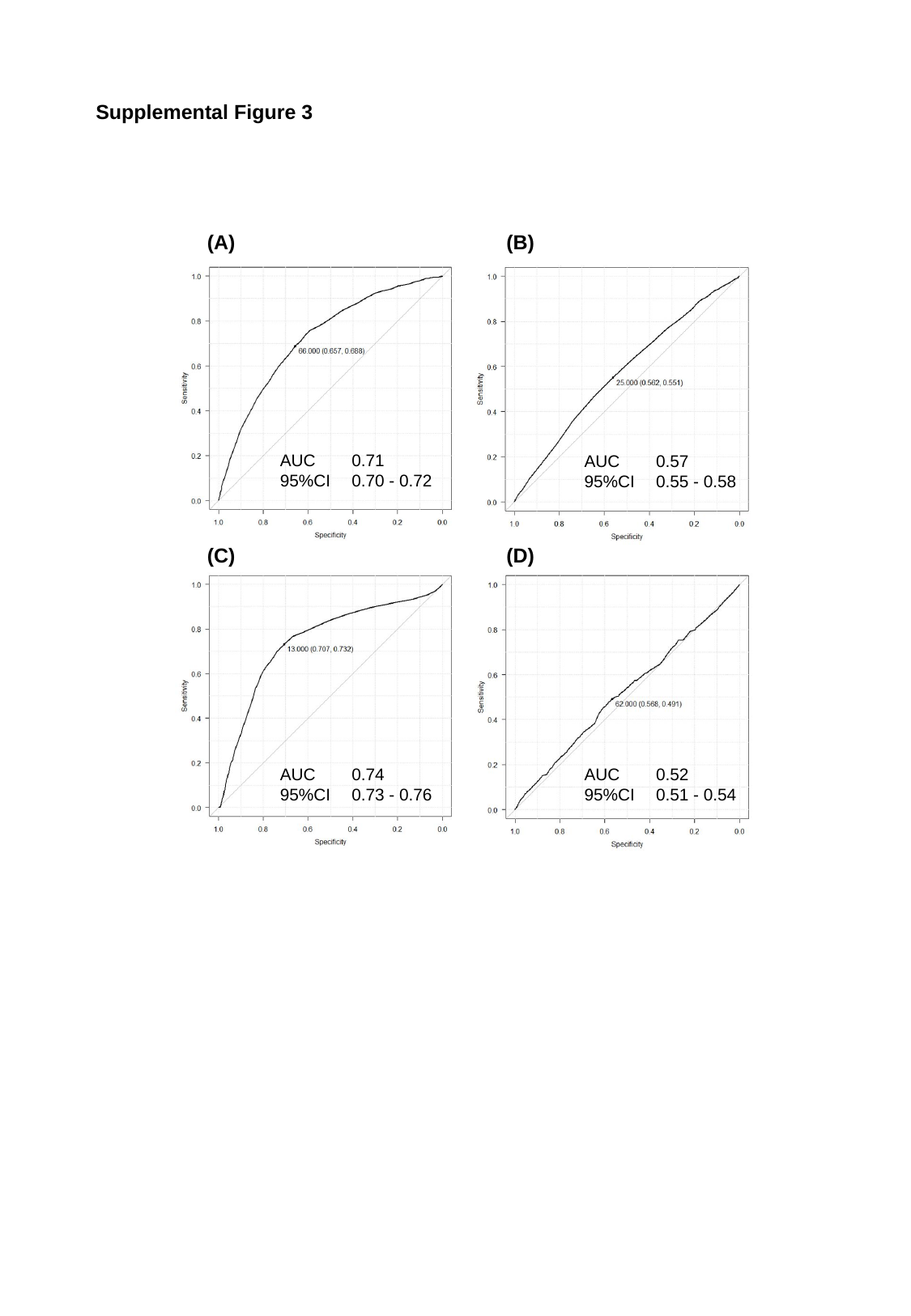

Supplemental Figure 3
(A)
(B)
AUC	0.71
95%CI	0.70 - 0.72
AUC	0.57
95%CI	0.55 - 0.58
(C)
(D)
AUC	0.74
95%CI	0.73 - 0.76
AUC	0.52
95%CI	0.51 - 0.54

Supplement: Supplementary file 4 — Additional file 4: Fig. S3. ROC curve analysis for predicting poor outcome in patients with severe COVID-19 receiving mechanical ventilation. (A) ROC curve for age, (B) for body mass index, (C) for the number of ventilator days, and (D) for the number of mechanical ventilations experienced at an institution for patients with severe COVID-19. ROC, receiver operating characteristic curve; COVID-19, coronavirus disease 2019 [file 13054_2022_4187_MOESM4_ESM.pptx]

## Slide 1
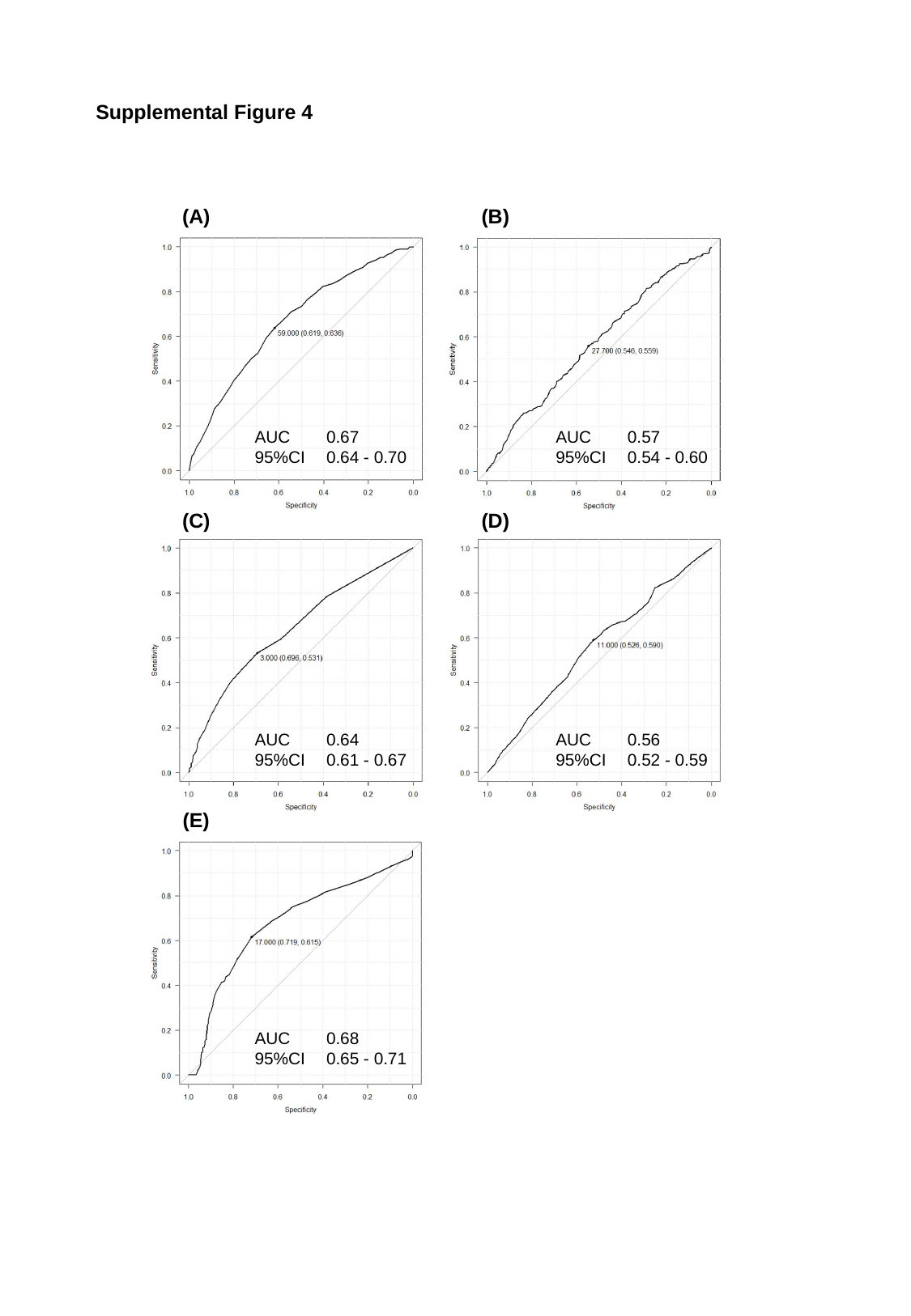

Supplemental Figure 4
(A)
(B)
AUC	0.67
95%CI	0.64 - 0.70
AUC	0.57
95%CI	0.54 - 0.60
(C)
(D)
AUC	0.64
95%CI	0.61 - 0.67
AUC	0.56
95%CI	0.52 - 0.59
(E)
AUC	0.68
95%CI	0.65 - 0.71

Supplement: Supplementary file 5 — Additional file 5: Fig. S4. ROC curve analysis for predicting poor outcome in patients with critical COVID-19 receiving ECMO. (A) ROC curve for age, (B) body mass index, (C) number of ventilator days before starting ECMO, (D) number of ECMO experiences at an institution for patients with critical COVID-19, and (E) number of ECMO days. ROC, receiver operating characteristic curve; COVID-19, coronavirus disease 2019; ECMO, extracorporeal membrane oxygenation [file 13054_2022_4187_MOESM5_ESM.pptx]
